# Characteristics, management and medical costs of patients with depressive disorders admitted in primary and specialised care centres in Spain between 2011 and 2016

**DOI:** 10.1371/journal.pone.0228749

**Published:** 2020-02-05

**Authors:** Josep Darbà, Alicia Marsà

**Affiliations:** 1 Universitat de Barcelona, Department of Economics, Barcelona, Spain; 2 BCN Health Economics & Outcomes Research S.L., Barcelona, Spain; Chinese Academy of Medical Sciences and Peking Union Medical College, CHINA

## Abstract

More than 10% of the population will suffer from a depressive disorder during their lifetime, which represents a substantial economic and social burden for healthcare systems and societies. Nonetheless, studies suggest that an important percentage of patients receive inadequate treatment. This study aimed to evaluate the characteristics of patients with depressive disorder in Spain, the current management of these disorders and the costs of specialised care. A retrospective multicentre study was designed including admission records from patients admitted due to a depressive disorder between 2011 and 2016, extracted from a Spanish claims database. The records obtained corresponded to 306,917 patients attended in primary care centres and 27,963 patients registered in specialised care settings. The number of admissions per patient progressively increased over the study period. A correlation was found with socioeconomic factors as the unemployment rate, increased versus the general population (OR = 1.41; 95%CI = 1.38–1.43). Equally, comorbid conditions as hypertension, disorders of lipoid metabolism, diabetes type II, other mood disorders and thyroid disorders were associated with severe presentations of a depressive disorder. In terms of disease management, patients with a severe disorder were the majority in specialised care settings, and most admissions were urgent and inpatient admissions. The use of both electroconvulsive therapy and drug therapy increased during the study period. In terms of costs, specialised care represented an annual cost of €9,654 per patient, and a total annual cost of €44,839,196. Altogether, improved detection and treatment protocols could contribute in reducing the burden that depressive disorders represent for the Spanish National Healthcare System.

## Introduction

It is estimated that 4.4% of the global population suffer from a depressive disorder, with more than 40 million people in Europe affected [1]. In Spain the estimated prevalence of major depressive disorder (MDD) was 8.0% among women and 4.1% among men in 2018 [2]; however, the average lifetime prevalence is estimated to reach the 10.6%, a percentage that is drastically increased in vulnerable groups as the hospitalised population [3, 4].

Mental illness represents substantial social and economic costs, as it is associated with disability and reduced quality of life [5–7]. The year 2016, these disorders were responsible for 6.43% of all disability-adjusted life-years (DALYs), and depression continues to be considered the leading cause of disability worldwide [8, 9]. Major depressive disorder alone summed about €155 million in direct medical costs in the region of Catalonia the year 2006, reaching the €735 million when lost productivity due to morbidity and mortality are considered [10].

Despite the significant social and economic burden of depressive disorders, several studies suggest that an important percentage of patients receive inadequate treatment and that the adherence to guidelines is seemingly low [11], although improved treatment protocols have been proven useful in decreasing the economic burden associated to these disorders [12].

The implementation of improved treatment protocols and specific guidelines is decisive for the optimisation of healthcare in mental illness. Treatment options include pharmacological therapy and physiological intervention, used in accordance with the severity of the symptoms and other factors and often through various stages of treatment [13]. Current clinical guidelines in Spain were conformed via the revision of multiple international guidelines and primarily based on the National Collaborating Centre for Mental Health (NCCMH) depression treatment and management guideline [14]. These guidelines specify diagnosis criteria and treatment options for the diverse presentations of depression, classifying treatment options in physiotherapy and drug therapy, and considering other options, as electroconvulsive therapy, for the treatment of severe disorders or treatment-resistant depression [15, 16]. Yet, the management of these disorders is seemingly not optimised, as data suggests that the percentage of patients receiving inadequate treatment is especially significant in primary care, which is the first level of care that meets the most basic health care needs, and around 71% of patients could seek help exclusively in primary care settings without being admitted in specialised care centres (hospitals) [11, 17].

This study aimed to evaluate the characteristics of patients with depressive disorders in Spain admitted in primary and specialised care centres, current disease management and the costs of specialised care, in an effort to provide a basis for the improvement of treatment guidelines for specialised care and, thus, the reduction of the burden related to these disorders.

## Methods

Admission records of patients admitted in primary and specialised care centres with a primary diagnosis of depression (admission motive) between 2011 and 2016 were extracted from a Spanish claims database, compiling data from public and private healthcare centres, in a retrospective multicentre study. The code P76 from The International Classification of Primary Care, second edition (ICPC-2) corresponding to depressive disorder was used to extract admission records from primary care facilities (Table 1). The 9th and 10th revisions of the International Statistical Classification of Diseases and Related Health Problems, Clinical Modification (ICD9-CM and ICD10-CM) codes were used to claim inpatient and outpatient care records from specialised care facilities (hospitals), corresponding to major depressive disorder with a single episode, recurrent depressive disorder and depressive disorders left unspecified; until 2015 data was codified using ICD9 codes while since 2016 ICD10 codes are used.

**Table 1:** Depressive disorders identified according to ICPC-2, ICD9-CM and ICD10-CM codes and number of admissions registered per each disorder.

Primary care	ICPC-2 (2011–16)		Admission number
Depressive disorder	P76		937,575
Specialised care	ICD9 (2011–15)	ICD10 (2016)	Admission number
**Major depressive disorder, single episode**	**296.2**	**F32**	**13,136**
Mild	296.21	F32.0	307
Moderate	296.22	F32.1	2,063
Severe, without mention of psychotic behaviour	296.23	F32.2	1,931
Severe, specified as with psychotic behaviour	296.24	F32.3	3,142
In partial or unspecified remission	296.25	F32.4	121
In full remission	296.26	F32.5	19
Unspecified	296.20	F32.9	5,553
**Recurrent major depressive disorder**	**296.3**	**F33**	**14,912**
Mild	296.31	F33.0	251
Moderate	296.32	F33.1	1,940
Severe, without mention of psychotic behaviour	296.33	F33.2	3,241
Severe, specified as with psychotic behaviour	296.34	F33.3	3,495
In partial or unspecified remission	296.35	F33.40/41	756
In full remission	296.36	F33.42	33
Unspecified	296.30	F33.9	5,196
**Depressive disorder, not elsewhere classified**	**311**	**F32/F33.8**	**8,201**

All admission files were used to analyse data on the nature of admissions, medical procedures and costs; for the analysis of patients’ characteristics solely the first admission registered per patient during the study period was included. Medical procedures were determined via ICD, Procedure Classification System (ICD9-PCS and ICD10-PCS) codes. To ensure consistency, tendencies in procedure utilisation were analysed solely via ICD9-PCS codes (2011–2015). Specialised healthcare costs were calculated based on the standardised average expenses of admissions and medical procedures determined by the Spanish Ministry of Health, which includes all expenses related to the admission: examination, medication, surgery, nutrition, costs associated to personnel, medical equipment and resources. Data related to the prescribed medication was not available.

The data extracted did not contain any parameters identifying healthcare centres and medical history, which are re-coded in the database maintaining records anonymised, in accordance with the principles of Good Clinical Practice and the Declaration of Helsinki. This research did not involve human participants and there was no access to identifying information; in this context the Spanish legislation does not require patient consent and ethics committee approval [18].

Data presentation is mainly descriptive. Descriptive values are presented in mean (SD). To assess the association with any socioeconomic factors odds ratio (OR) with 95% confidence interval (CI) were used, with the total population attended in primary care as reference group. The population without mention of severe MDD was used as a reference when indicated [19]. Statistical analyses were performed using Microsoft Excel Professional Plus 2010 (Microsoft Corporation, Redmond, WA, USA).

## Results

### Primary care

The 937,575 admissions registered in primary care corresponded to 306,917 patients. In a temporal analysis, a progressive increase in the number of admissions per patient was observed, up to the 6.69 admissions per patient registered in 2016.

Overall, 71.9% of the patients were females, with an average age of 57.69 years (SD = 17.92). Male patients were slightly younger averaging 54.83 years (SD = 17.80).

Socioeconomic data extracted from primary care records revealed an increased presence of patients with an annual income level under the €18,000, 77.43% of patients with a specified income level belonged to this group (OR = 1.03; 95%CI = 1.02–1.03); 20.92% of patients were between the €18,000 and the €99,999 (OR = 0.89; 95%CI = 0.88–0.90). The global unemployment rate throughout the study period was also increased versus the general population (OR = 1.41; 95%CI = 1.38–1.43).

### Specialised care

**Fig 1:**
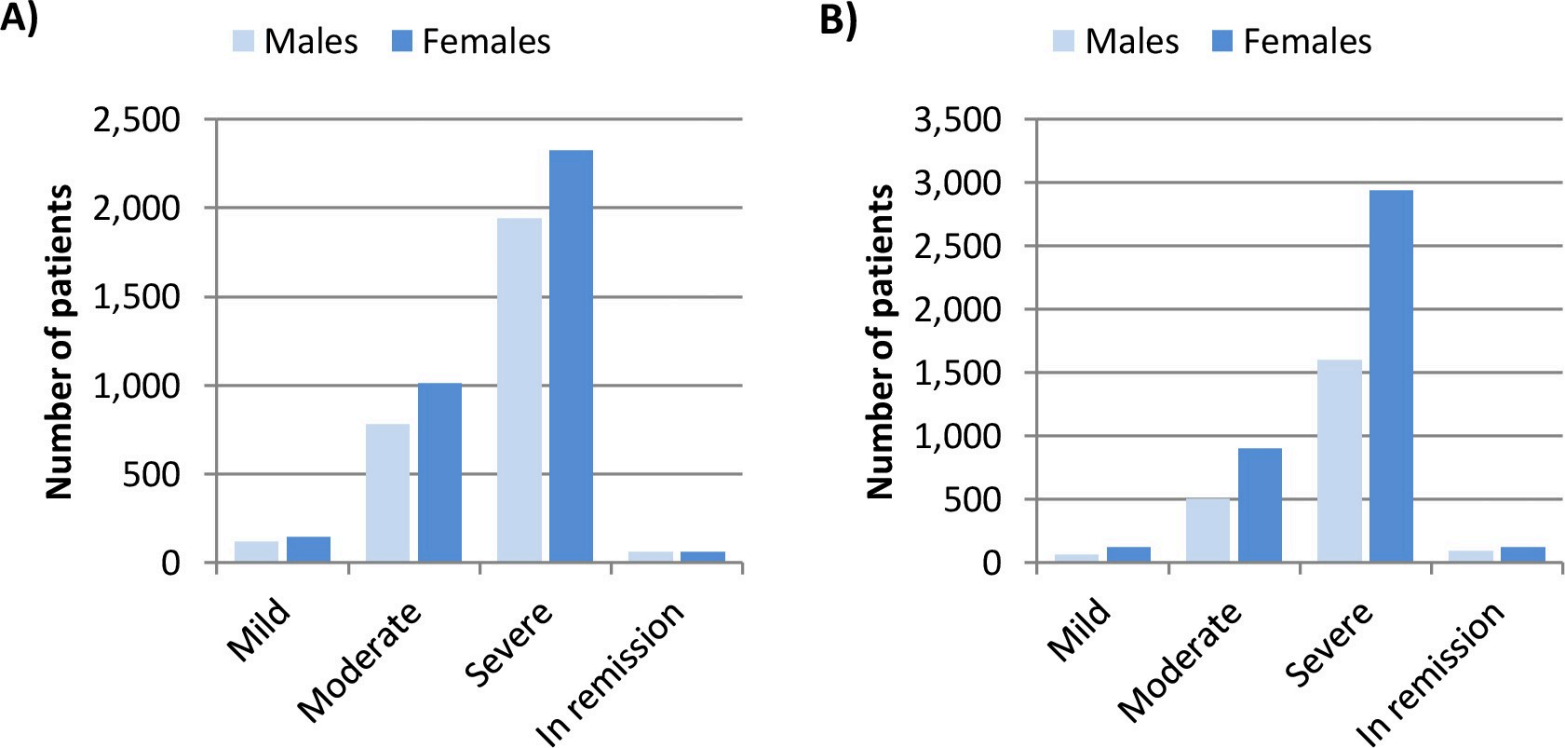
Number of male and female patients admitted in specialised care with: A) a single episode MDD or B) a recurrent MDD.

Admission records corresponded to 27,963 patients attended in specialised care settings; 11,107 were admitted with a single episode major depressive disorder and 9,647 with recurrent major depressive disorder. In addition, 7,209 patients were registered with an unspecified depressive disorder. Most of the patients attended in specialised care with a specified disorder were labelled as severe (Fig 1). According to ICD classification, when considering the complete patients’ history, 33.22% of patients entered remission at some point during the study period.

Herein, 59.97% of patients admitted with a depression disorder were females, a smaller percentage than in primary care; yet, female patients were more than 50% in all groups with a specified disorder.

The mean age of patients attended in specialised care was 52.70 years (SD = 17.94); no relevant fluctuations were found over the years. Equally, male and female patients displayed, on average, a similar age, although males with a single episode MDD in remission were younger.

Primary diagnosis or admission motive was, in all cases, a depressive disorder; however, specialised care records included secondary diagnoses registered during the admission. Hypertension and disorders of lipoid metabolism (hypercholesterolaemia, hyperlipidaemia and hyperglyceridaemia) were the most common conditions (Table 2). Several conditions were more frequent in patients with severe MDD versus those without severe MDD, as hypertension, diabetes, lipoid disorders and thyroid disorders. The frequency of suicidal ideation and suicide attempts was also increased in this group, although the difference versus the patients without severe MDD was relatively small. Contrarily, drug abuse was registered in 4.26% of patients with severe MDD, 4.93% of those without MDD. When analysed independently, patients that attempted suicide displayed similar secondary diagnoses, but a higher rate of alcohol abuse (17.61%; OR = 1.64; 95%CI = 1.42–1.90).

**Table 2:** Secondary diagnoses found in more than 5% of all patients admitted with or without severe MDD.

Secondary diagnoses	% Severe MDD	% Without severe MDD	Odds Ratio ^a^ (95%CI)
**Unspecified essential hypertension**	29.59	17.68	1.96 (1.85–2.10)
**Suicidal ideation**	17.89	16.60	1.10 (1.01–1.19)
**Disorders of lipoid metabolism**	21.70	13.83	1.73 (1.60–1.86)
**Tobacco use disorder**	15.39	13.84	1.13 (1.04–1.23)
**Alcohol abuse**	9.63	11.08	0.85 (0.77–0.95)
**Diabetes mellitus type II**	14.49	8.98	1.72 (1.57–1.88)
**Other mood disorders**	8.81	7.73	1.15 (1.04–1.29)
**Thyroid disorders**	10.24	6.44	1.66 (1.49–1.84)
**Suicide**	6.29	4.93	1.29 (1.14–1.47)

^a^ Odds ratio for diagnosis frequency, patients with severe MDD vs. without severe MDD.

In addition, records offered a clear view of disease management. The 86.64% of all specialised care admissions were urgent; 414 admissions corresponded to outpatient consultations versus 35,835 inpatient admissions. In this last case, mean length of hospital stay was 16.90 days, 22.80 days in patients with severe MDD and 14.05 days in those without severe MDD. Readmission rate within 30 days was 8.12% in patients with a single episode disorder and 16.99% in patients with a recurrent disorder, while it was 15.63% and 14.04% in patients with severe MDD and those without mention of severe MDD respectively. The service to discharge the most patients was psychiatry (91.78%).

**Fig 2:**
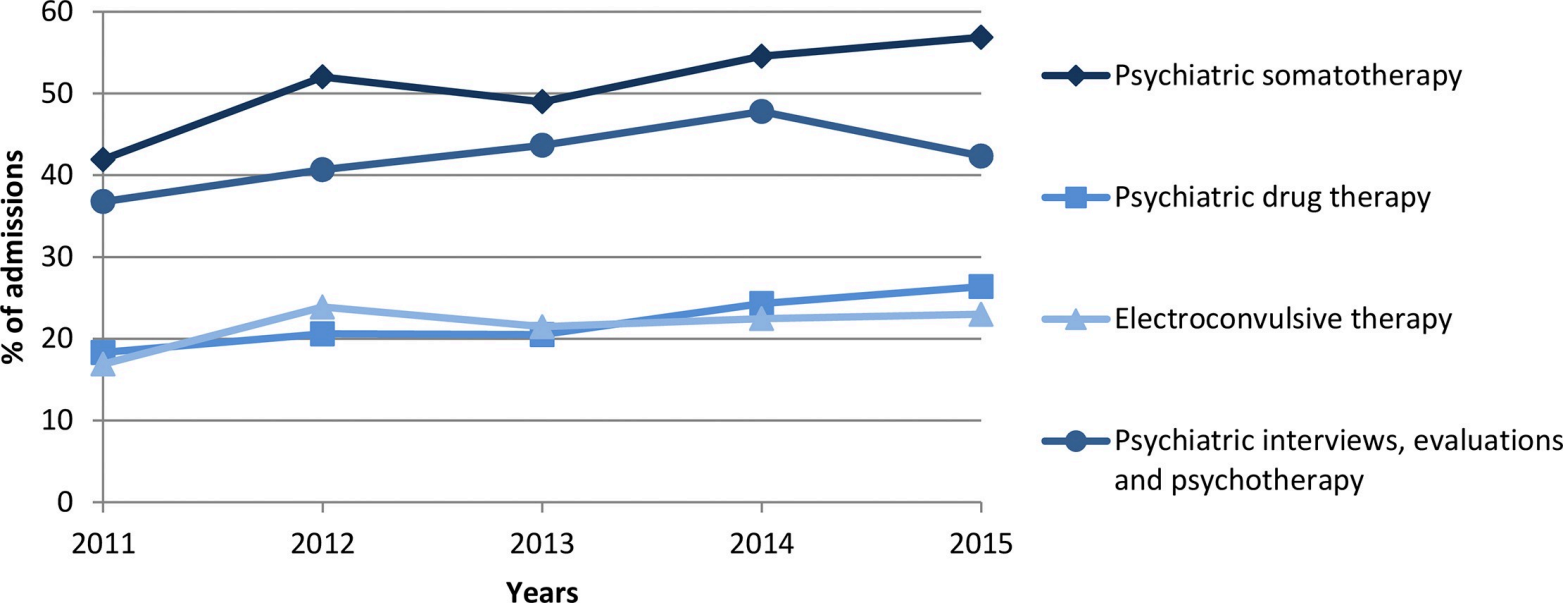
Changes registered in procedure utilisation over time (2011–2015).

The most common medical procedures could be classified in several categories, according to their ICD9-PCS and ICD10-PCS codes, as: psychiatric somatotherapy, psychiatric interviews, consultations and evaluations, individual psychotherapy, referral for psychological rehabilitation, psychological evaluation and testing and general diagnostic tests and imaging (Table 3). Patients with severe MDD registered higher rates of drug therapy (OR = 3.72; 95%CI = 3.51–3.94), while such difference was not registered in the use of electroconvulsive therapy. Overall, psychiatric somatotherapy was the most common therapy for all patients and its use increased from the 41.93% to the 56.87% between 2011 and 2015 due to the increase of both electroconvulsive therapy and drug therapy (Fig 2).

**Table 3:** Medical procedures found in more than 1% of admissions of all patients admitted with or without severe MDD.

Medical procedure	% Severe MDD	% Without severe MDD	Odds Ratio ^a^ (95%CI)
**Psychiatric somatotherapy**	70.95	41.64	3.42 (3.25–3.60)
Electroconvulsive therapy	22.87	21.81	1.06 (1.00–1.13)
Psychiatric drug therapy	37.67	13.97	3.72 (3.51–3.94)
Neuroleptic therapy	8.50	5.11	1.72 (1.57–1.90)
**Psychiatric interviews, consultations, evaluations**	**32.45**	**31.02**	**1.07 (1.01–1.13)**
Other psychiatric interview and evaluation	14.20	15.33	0.91 (0.85–0.98)
Psychiatric mental status determination	12.26	9.87	1.28 (1.18–1.38)
Psychiatric commitment evaluation	4.37	4.37	1.00 (0.89–1.13)
**Individual psychotherapy**	**7.39**	**10.12**	**0.71 (0.65–0.77)**
Other psychotherapy and counselling	3.55	5.96	0.58 (0.51–0.66)
Other individual psychotherapy	3.10	4.38	0.70 (0.61–0.80)
Supportive verbal psychotherapy	1.44	2.54	0.56 (0.46–0.68)
Crisis intervention	1.96	2.11	0.93 (0.78–1.10)
**Referral for psychological rehabilitation**	**1.95**	**2.65**	**0.73 (0.62–0.86)**
Referral for psychiatric aftercare	1.88	2.24	0.83 (0.70–0.99)
**Physiological evaluation and testing**	**2.05**	**1.48**	**1.39 (1.16–1.67)**
**Diagnostic tests**	-	-	-
Microscopic examination of blood/urine	15.34	18.45	0.80 (0.75–0.86)
Diagnostic imaging of head and brain	22.09	15.00	1.61 (1.51–1.71)
Diagnostic imaging of chest and abdomen	8.08	9.75	0.81 (0.75–0.89)
Diagnostic ultrasound of heart/ electrocardiogram	7.60	8.77	0.86 (0.78–0.94)
Injection of antibiotic or therapeutic substance	4.20	4.68	0.89 (0.79–1.00)

^a^ Odds ratio for diagnosis frequency, patients with severe MDD vs. without severe MDD.

Finally, admission records were associated to a cost per admission, providing an approximation to the direct medical cost of depressive disorders. Altogether, each patient represented an average annual cost of €9,654 for the healthcare system, considering that 96.28% of patients were financed by the social security system. In patients with a recurrent MDD, this cost increases to €9,947. Equally, patients with severe MDD represented an annual cost of €10,883 per patient, whereas this figure in patients without severe MDD was €9,094.

**Fig 3:**
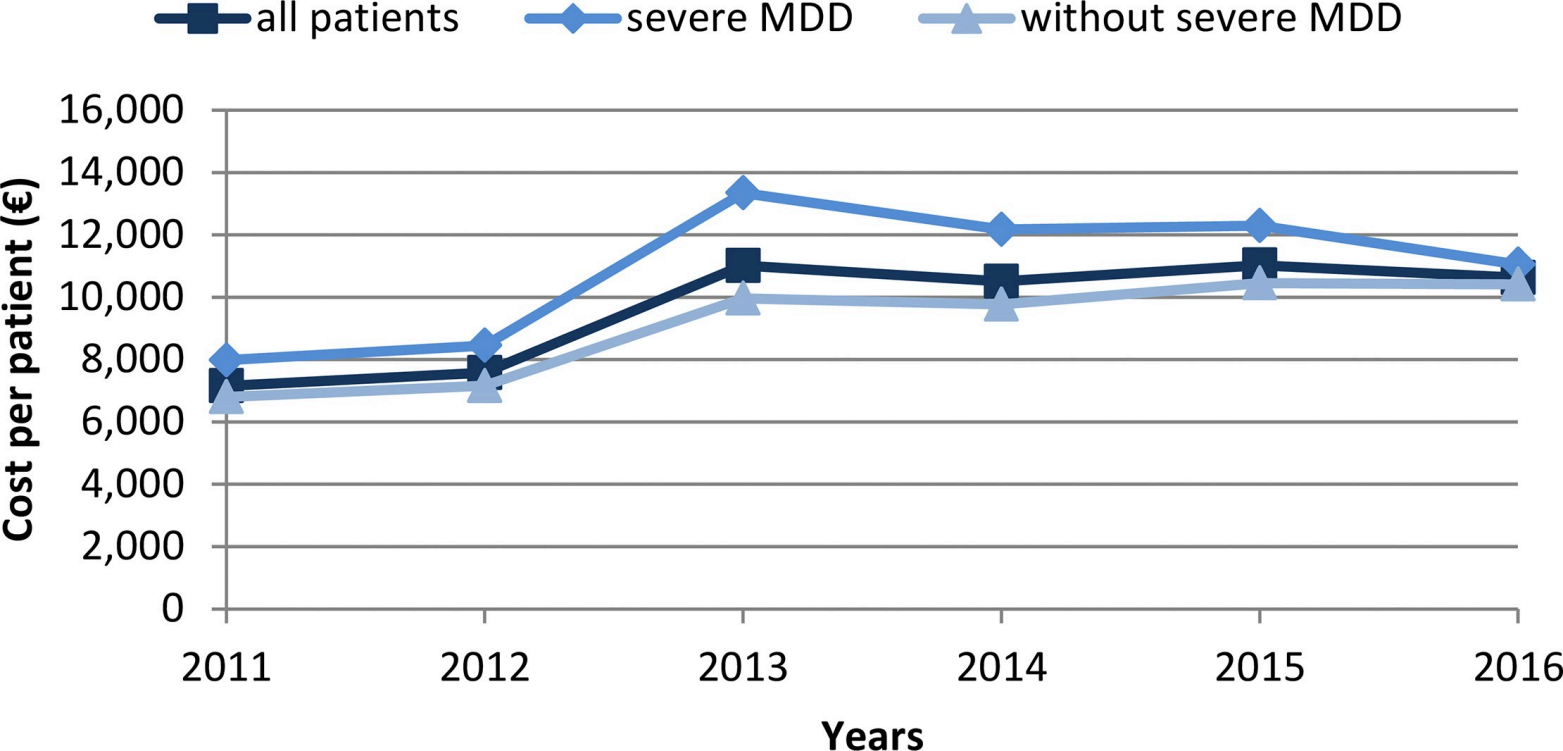
Annual medical costs of specialised care of depressive disorders considering all patients, patients with MDD and patients without MDD.

When analysed per years, the progressive increase in the number of admissions is visible as a gradual increase of healthcare costs, especially noticeable between the years 2012 and 2013 (Fig 3). Nonetheless, the cost of patients with MDD peaks the year 2013 and slightly decreases the following years.

With all admissions considered, the annual cost of specialised care of depressive disorders in Spain was €44,839,196.

## Discussion

The high prevalence and substantial social and economic burden of depressive disorders contrasts with the insufficient detection of these disorders in primary care and the inadequate treatment received by many patients. This study describes the current status of depressive disorders in Spain, in terms of patient characteristics, disease management and its costs.

The patient profile described in this study is comparable to previous evaluations. The increased percentage of female patients in primary care settings has been previously described in Spain [11, 20]. On average, the patients that access specialised care are slightly younger and are mostly diagnosed with a severe disorder, which is not surprising given the low detection rate of mild forms of depression [21].

The association of depressive disorders with socioeconomic indicators is apparent in patients’ unemployment rate, increased versus the general population. The portion of patients with an income level under €18,000 was also increased, strengthening the evidence linking these factors with depression [22, 23]. All evidence suggests that detection protocols should consider socioeconomic indicators to establish risk groups, a practice that is currently not into consideration in Spain [15, 16]. Equally, the presence of certain comorbid conditions found in patients with depressive disorder and, at a higher rate, in patients with a severe disorder should be taken into account, particularly those described in previous assessments, as are the increased levels of triglycerides and cholesterol or the presence of type II diabetes [24, 25]. Additionally, there is strong evidence linking thyroid disorders, especially hypothyroidism, with depression [26, 27], which herein correlate with the severity of the MDD. Alcohol abuse appeared associated to suicide and should be considered one of the risk factors for suicide in patients with depression also within the Spanish population [28].

Currently, most patients with depressive disorders in Spain are attended in primary care, as considering the number of admissions registered in primary care settings versus specialised care. The number of admissions per patient increased during the study period in primary care, which indicates the need for successive consultations. In specialised care, most of the admissions were urgent and hospitalisation was required. Interestingly, the use of electroconvulsive therapy increased over time in parallel with drug therapy, although it is only recommended for the treatment of severe and resistant disorders [15]. A study from 2012 based on a questionnaire showed that 84.2% of psychiatric units prescribed electroconvulsive therapy, a percentage that grew considerably since 2001, with great variation among Spanish regions [29]. These data indicated the need to unify criteria for the application of this therapy alternative, while current recommendations focus on the flexibility and adaptability of its application to reduce side effects [30], still, psychotherapy is preferred in the mildest cases of the disorder [15, 16].

In terms of costs, evaluations at the European level estimated a cost of specialised care that represented 8.5% of all direct medical costs, while primary care summed 18.6% of the costs and pharmacological care the 7.6% [5]. Herein, specialised care summed €44,839,196 per year, costs that, added to primary and pharmacological care, could increase to about €529 million. In addition, the productivity losses due to disability and mortality contribute to increase the burden of depressive disorders [8, 9]. More effective detection and treatment protocols, especially in primary care and for patients with recurrent MDD, could contribute in reducing this burden. Our analysis of annual costs and their evolution suggests a decrease in the annual cost per patient after the year 2013, noticeable for patients with severe MDD, which could be indicative of changes in the in-hospital treatment that these patients receive.

The conclusions of this study were subjected to a series of limitations. Firstly, the costs of primary care and prescribed medication could not be directly calculated via this database. Secondly, primary care records do not include disease management data, which impedes a more consistent analysis of treatment preferences and deficiencies, particularly in patients with mild disorders. Finally, data obtained in this study should be considered in the context of characterising the population that is hospitalised due to MDD and is not translatable to the entire population affected with depression that might present different associated conditions.

## Conclusions

Depressive disorders continue to represent a substantial burden for the Spanish National Healthcare System, reaching the €44,839,196 solely in specialised healthcare. The improvement of treatment protocols, taking into account socioeconomic risk factors and the presence of associated conditions, should contribute in reducing this burden while focusing in a more flexible and personalised treatment. In particular, depression management in primary care centres should be optimised as these settings attend the major portion of patients.
